# Efficient Accumulation of Amylopectin and Its Molecular Mechanism in the Submerged Duckweed Mutant

**DOI:** 10.3390/ijms24032934

**Published:** 2023-02-02

**Authors:** Yu Liu, Ruiting Yan, Zonghao Li, Shusheng Fan, Chuantong Li, Ruikang Yu, Huaqing Liu, Yingzhen Kong, Haimei Li, Xianfeng Tang, Gongke Zhou

**Affiliations:** 1 College of Landscape Architecture and Forestry, Qingdao Agricultural University, No. 700 Changcheng Road, Chengyang District, Qingdao 266109, China; 2 Academy of Dongying Efficient Agricultural Technology and Industry on Saline and Alkaline Land in Collaboration with Qingdao Agricultural University, Dongying 257000, China; 3 College of Resources and Environment, Qingdao Agricultural University, Qingdao 266109, China; 4 College of Agronomy, Qingdao Agricultural University, Qingdao 266109, China

**Keywords:** *Lemna*, amylopectin, physical property, metabolome, transcriptome

## Abstract

Large-scale use of fossil fuels has brought about increasingly serious problems of environmental pollution, development and utilization of renewable energy is one of the effective solutions. Duckweed has the advantages of fast growth, high starch content and no occupation of arable land, so it is a promising starchy energy plant. A new submerged duckweed mutant (*sub-1*) with abundant starch accumulation was obtained, whose content of amylopectin accounts for 84.04% of the starch granules. Compared with the wild type (*Lemna aequinoctialis*), the branching degree of starch in *sub-1* mutant was significantly increased by 19.6%. Chain length DP 6–12, DP 25–36 and DP > 36 of amylopectin significantly decreased, while chain length DP 13–24 significantly increased. Average chain length of wild-type and *sub-1* mutant starches were greater than DP 22. Moreover, the crystal structure and physical properties of starch have changed markedly in *sub-1* mutant. For example, the starch crystallinity of *sub-1* mutant was only 8.94%, while that of wild-type was 22.3%. Compared with wild type, water solubility of starch was significantly reduced by 29.42%, whereas swelling power significantly increased by 97.07% in *sub-1* mutant. In order to further analyze the molecular mechanism of efficient accumulation of amylopectin in *sub-1* mutant, metabolome and transcriptome were performed. The results showed that glucose accumulated in *sub-1* mutant, then degradation of starch to glucose mainly depends on α-amylase. At night, the down-regulated β-amylase gene resulted in the inhibition of starch degradation. The starch and sucrose metabolism pathways were significantly enriched. Up-regulated expression of *SUS*, *AGPase2*, *AGPase3*, *PYG*, *GPI* and *GYS* provide sufficient substrate for starch synthesis in *sub-1* mutant. From the 0H to 16H light treatment, granule-bound starch synthase (*GBSS1*) gene was inhibited, on the contrary, the starch branching enzyme (*SBE*) gene was induced. Differential expression of *GBSS1* and *SBE* may be an important reason for the decrease ratio of amylose/amylopectin in *sub-1* mutant. Taken together, our results indicated that the *sub-1* mutant can accumulate the amylopectin efficiently, potentially through altering the differential expression of *AGPase*, *GBSS1, SBE,* and *BAM*. This study also provides theoretical guidance for creating crop germplasm with high amylopectin by means of synthetic biology in the future.

## 1. Introduction

Duckweed is one kind of monocot aquatic plant that grows on the water surface. It has the characteristics of fast reproduction, high starch content and low lignin content [1]. Therefore, duckweed has been considered as a starchy energy plant for many years. Duckweed has a simple structure, consisting of only two parts: Frond and root [1]. The shape and size of fronds vary with species and growth environment. For example, the fronds of *Lemna aequinoctialis* is symmetrical and is nearly round or obovate in shape. Frond is the main organ where carbon dioxide (CO_2_) in the atmosphere is absorbed to synthesize carbohydrates and store energy in the form of starch. However, the storage site of starch in frond is not well understood.

Starch is an important natural glucose polymer, consisting of amylose and amylopectin. In general, the former is connected by α-(1,4) glycosidic bonds, and also linked by α-(1,4) glycosidic bond within the side chains, however the side chains are connected to the main chain or intercepted by α-(1,6) glycosidic bond. The latter is connected end to end by α-(1,4) glycosidic bonds, and at the branch chain connected by α-(1,6) glycosidic bonds. Amylopectin is the main type of polysaccharide, and its content varies greatly, generally ranging from 72% to 85% in starch granule [2,3]. Amylopectin determines the physicochemical properties of starch particles. For example, amylopectin has a higher molecular weight, the peak value of amylopectin chain length distribution was 10^8^ Da, while that of amylose was 10^6^ Da [4]. Amylopectin contains more branch chains than amylose, and these branch points cluster together to form a homogenous allotrope. For example, cereal starch is A type homogenous allotrope and is commonly found in corn, wheat, rice, and barley [5,6,7,8]. Rhizome crop starch is B type homologous allotropes and is commonly found in potato and cassava [5,9]. From the perspective of evolution, the physicochemical properties and internal structure of starch are highly conserved. A comparative study on the physicochemical properties of starch was performed between *Spirodela oligorrhiza* and *Lemna minor*. We found that the amylopectin content was 79.15% and 72.77%, respectively, and the average chain length was higher than DP 28 [10]. Meanwhile, a typical B-type diffraction pattern in *Spirodela oligorrhiza* and *Lemna minor* were detected by wide-angle XRD [10]. Recently, a new submerged duckweed mutant (*sub-1*) was obtained in our laboratory. The total starch content of *sub-1* mutant was twice that of the wild type, and the released glucose and ethanol yield were significantly increased [11]. However, the physicochemical properties and internal structure of starch were not illustrated in this new variety (*sub-1*).

In plant, starch metabolism has been relatively well understood, which involve four types of enzymes [12,13]. AGPase is the first limiting enzyme, which catalyzes the synthesis of ADP-glucose by glucose-1-phosphate and ATP in the starch synthesis pathway [12]. ADP-glucose is the substrate for the second limiting enzyme, including granule-bound starch synthase (GBSS) and soluble starch synthase (SSs). GBSS and SSs transfer the glucose group of ADP-glucose to the non-reducing end of the (1,4)-glucan chain to form an α-(1,4) glycosidic bond. Starch branching enzymes (SBEs) catalyze the cleavage of 1, 4-glycosidic bond, and link the released oligosaccharide chain to the C6 hydroxyl group of the glucan chain residue to form (1,6)-glycosidic bond and to produce the branched chain [12]. Debranching enzymes (DBEs) are the last step of starch synthesis, which hydrolyze the α-(1-6) glucoside bond in the glucoside chain to achieve the rearrangement of polysaccharide molecules. Two types of DBEs have been identified, including isoamylase (ISA1 and ISA2) and pullulanase (PUL). In the stage of starch degradation, firstly, glucan-water dikinase (GWD) and phosphoglucan-water dikinase (PWD) phosphorylate starch granules. Subsequently, a portion of the loose starch granules were degraded by β-amylase (BAM) to produce maltose, and then under the action of disproportionating enzyme (DPE1) to produce glucose [14]. Meanwhile, another portion of the loose starch granules were degraded by α-amylase to produce malto-oligo saccharides, and then under the action of BAM and DPE1 to produce glucose. While the starch synthesis and degradation pathway has been roughly clear in plant. However, studies on the key enzyme genes of starch metabolism in duckweed are relatively limited, except for AGPase [15,16].

A new duckweed variety with high starch was screened from the heavy ion radiation mutagenic mutant library. As it accumulated a large amount of starch and sank to the bottom of water under oligotrophic or crowded conditions, we named it *sub-1* mutant [11]. There is no need for manual screening and collection, which can greatly reduce the cost of bioethanol production and damage to the environment. Therefore, the new variety *sub-1* is a quality raw material for bioethanol production. In this study, we analyzed the internal structure and physiochemical characteristics of starch particles in *sub-1* mutant, including chain length distribution, branching degree, crystallinity, XRD diffraction and so on. Metabolome and transcriptome investigate the differentially accumulated metabolites and differentially expressed genes in the starch metabolism pathway and explore the transcription factors that may affect starch accumulation. This study laid a theoretical foundation for the development and utilization of *sub-1* mutant in the future. 

## 2. Results

### 2.1. Sugar and Starch Were Massively Accumulated in sub-1 Mutant

**Figure 1:**
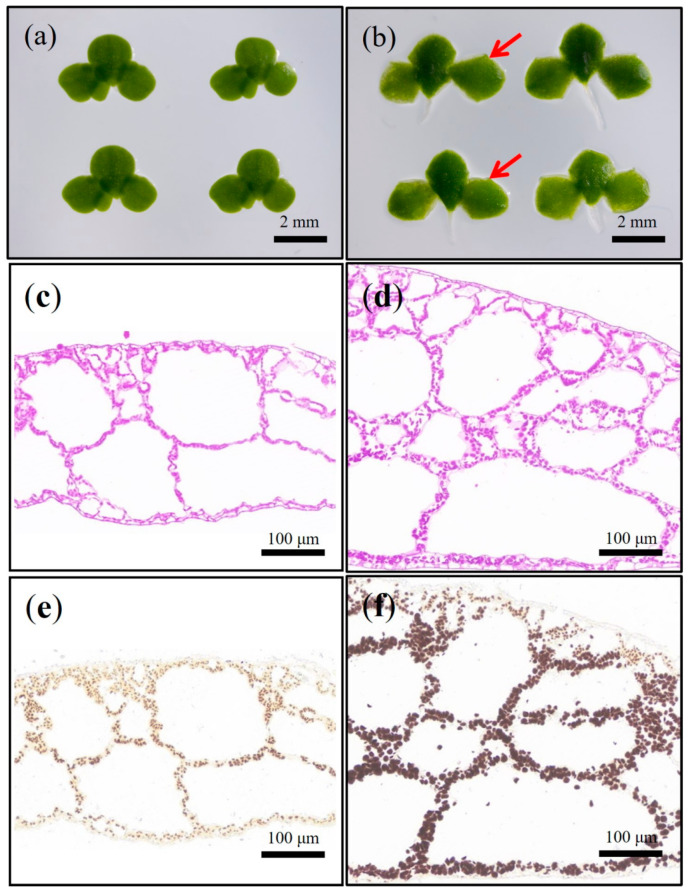
The analysis of phenotypic characteristics of the *sub-1* mutant. (**a**,**b**): Frond shape difference between wild type and *sub-1* mutant. (**c**,**d**): PAS staining of wild type and *sub-1* mutant. (**e**,**f**): I_2_/KI staining of wild type and *sub-1* mutant. Scale bars = 2 mm, 100 μm.

The frond traits of wild type (*L. aequinoctialis* 6002) appeared to be obovate (Figure 1a), while those of the *sub-1* mutant were square (Figure 1b). Then, paraffin sections of fronds were prepared and I_2_/KI staining was used to analyze the starch accumulation. As shown in Figure 1, the fronds of wild-type and *sub-1* mutant were different in color, dark brown in *sub-1* mutant (Figure 1f) and light brown in wild type (Figure 1e). Compared with wild-type, the volume and quantity of starch granules in *sub-1* mutant increased significantly (Figure 1f). Starch granules were mainly distributed in the inner cavity and lower epidermal cells, while not found in the upper epidermal cells. It was unexpectedly found that the fronds had an additional layer of aerenchyma, resulting in a significant 72.49% increase in leaf thickness in *sub-1* mutant compared to wild type (Figure S1). The above result indicated that the *sub-1* mutant accumulated a large amount of starch particles, inner cavity and lower epidermal cells were the main storage sites of starch, especially the additional layer of cells in the middle of the fronds. Sugar and starch were dyed purplish-red by PAS solution. Compared with wild type, the *sub-1* mutant showed deep purplish-red all over the fronds, reflecting the accumulation of large amounts of sugar and starch (Figure 1c,d). Based on the results of I_2_/KI and PAS staining, we concluded that the upper epidermal cells of frond were the main sites of sugar metabolism, and the inner cavity cells were used for starch storage. Starch granules were extracted from wild type and *sub-1* mutant, and amylose content of them were detected. Compared with wild type, the content of amylose obviously decreased by 34.43%, while that of amylopectin significantly increased by 11.08% in *sub-1* mutant (Table 1). The ratio of amylose to amylopectin was 0.32 and 0.19 in wild-type and *sub-1* mutant, respectively (Table 1). It was particularly noted that the proportion of amylopectin in *sub-1* mutant was as high as 84.04% (Table 1). Therefore, we conducted follow-up experiments on the physicochemical properties and efficient accumulation of amylopectin in *sub-1* mutant.

### 2.2. Chain Length Distribution and Branching Degree of Amylopectin

**Figure 2:**
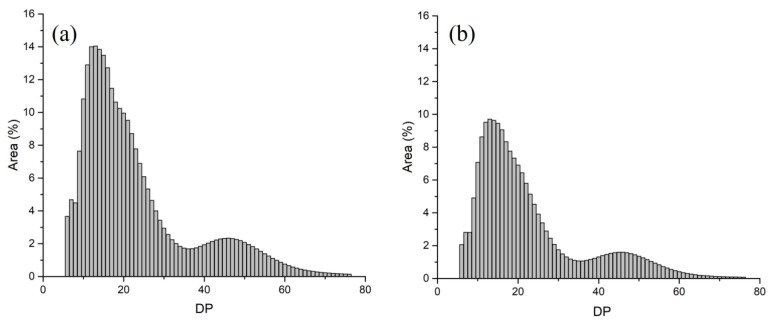
Chain length distributions of amylopectin in the wild type (**a**) and *sub-1* mutant (**b**).

Starch is a polysaccharide formed by the polymerization of glucose molecules. The number of glucose molecules involved in the composition of starch is called the degree of polymerization (DP). With the value of DP as the horizontal coordinate and the relative peak area of DP as the vertical coordinate, the distribution curves of chain lengths can be obtained. According to the structure model of amylopectin, the relative number of amylopectin chain lengths are normalized and divided into four parts: DP 6–12, DP 13–24, DP 25–36 and DP > 36, corresponding to A, B1, B2, and B3 chains [17]. In the wild-type duckweed, the ratios of these four parts were 21.26%, 47.30%, 14.02% and 17.42%, respectively (Figure 2a, Table 2). In *sub-1* mutant, the ratios of these four parts were 20.78%, 49.50%, 13.02% and 16.70%, respectively (Figure 2b, Table 2). Compared with wild-type, the degree of polymerization at DP 6–12, DP 25–36 and DP > 36 decreased clearly, while DP 13–24 increased significantly in *sub-1* mutant. In addition, the average chain lengths of amylopectin in wild type and *sub-1* mutant were 23.16 and 22.72, respectively (Table 2). The degree of branching is an important index to evaluate the amylopectin. Compared with wild-type, the branching degree of starch in *sub-1* mutant significantly increased by 19.6% (Table 3). This result indicated that starch of *sub-1* mutant has more branches (i.e., 1, 6-glycosidic bond form).

### 2.3. X-ray Diffraction (XRD) Analysis

**Figure 3:**
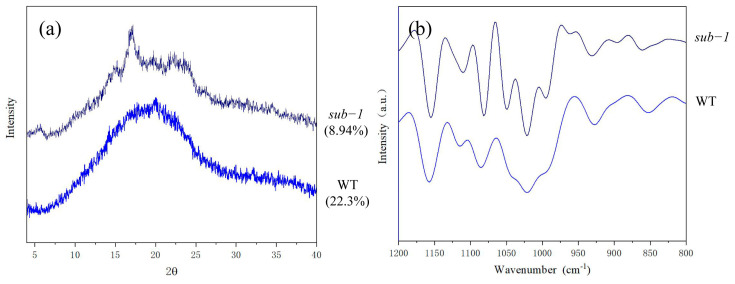
X−ray diffraction patterns (**a**) and fourier transform infrared spectroscopy (**b**) of the two duckweed starchs.

The crystallinity and crystal morphology of starch were analyzed by X-ray diffraction. The results showed that the starch crystallinity of wild-type was 22.3%, while that of the *sub-1* mutant was only 8.94% (Figure 3a). Previous research indicated that short chains of amylopectin (DP 10–20) can form a double helix and participate in the crystal region formation of starch granule [18]. When short chains were fixed in the long chains located in the non-crystalline lamellar of starch granule, the crystallinity decreases [18]. Therefore, we speculated that the ratio of short chain (DP 6–12) decreased, meanwhile medium-long chain (DP 13–24) were fixed in the non-crystalline lamellar, leading to the decrease in the crystallinity of starch in *sub-1* mutant. It has been reported that B-type starch has the peaks at diffraction angle (2θ) of 5.6°, 17°, 22°, and 24°. V-type starch has diffraction peaks at 7.8°, 12.5° and 19.5°, which is composed of a single helical structure and co-exist with other crystal types [19]. We found that the peak at diffraction angle (2θ) of around 20° was indicative of V-type pattern in wild-type, which implies that starch granules form amylose-lipid complexes [19]. In *sub-1* mutant, starch granules showed diffraction peaks at 5° (0.6), 15° (0.6), 17.5° (5.6), 20° (2.8) and 23.5° (3.3), reflecting the typical characteristics of B-type starch. The long chain of amylopectin maintains the rigidity of starch granule and also maintain the long-range order of starch granule [2,20]. The above results indicated that the reduced long chain of amylopectin affect the long-range order within the starch granules, and crystal morphology of starch granules change from V-type to B-type in *sub-1* mutant.

### 2.4. Fourier Transformed Infrared Spectrometry (FTIR) Analysis

The degree of short-range order of starch can be analyzed by far infrared scanning techniques, especially the alteration of the double helix structure. 1200 cm^−1^ to 800 cm^−1^ are mostly from the elastic vibrations of C-O and C-C, and this range is sensitive to the physiological state of starch [21,22]. Among them, 1047 cm^−1^ and 1022 cm^−1^ represent the crystalline and amorphous regions, respectively, and the absorption peak at 995 cm^−1^ corresponds to the bending vibration of the hydroxyl group [23]. The ratio of 1047/1022 mainly characterized the degree of short-range order (DO). The ratio of 1022/995 mainly characterized the double helix order (DD). Infrared spectrometry showed that the ratio of 1047/1022 was lower and the 1022/995 was higher, indicating that the degree of short-range order was low in *sub-1* mutant (Figure 3b). This result contradicts the high amylopectin content of the *sub-1* mutant, and the underlying molecular mechanism needs further investigation.

### 2.5. Physicalproperty of Starch in sub-1 Mutant

Water solubility and swelling power reflect the interaction between starch and water [24]. Compared with wild type, swelling power of starch was significantly increased by 97.07%, while the water solubility of starch was significantly decreased by 29.42% in *sub-1* mutant, implying the existence of a strong interaction between crystalline and amorphous regions (Table 3). Studies have shown that swelling power was negatively correlated with the amylose content [25]. The longer branched chain of amylopectin, the lower swelling power [26]. In this study, swelling power was positively correlated with amylopectin content, especially that of medium-long chain (DP 13–24), and negatively correlated with short chain (DP 6–12) and long chain (DP > 36) amylopection in *sub-1* mutant.

### 2.6. Metabolome Analysis

**Figure 4:**
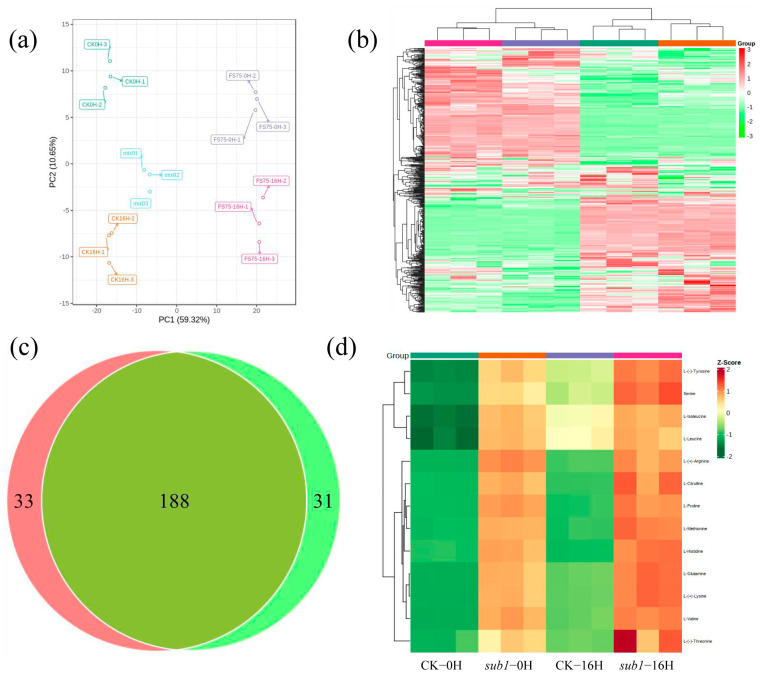
The quality of metabolome and differentially accumulated metabolites analysis. (**a**) PCA analysis, (**b**) heatmap of differentially accumulated metabolites, green represents down-regulated, red represents up-regulated, (**c**) Venn diagram analysis, (**d**) heatmap of differentially accumulated amino acids, green represents down-regulated, and red represents up-regulated.

Starch is synthesized in the light and degraded into sugars during the night for plant growth and development. To explore the effect of starch accumulation in the metabolites such as sugars, metabolome was performed under the condition of 0H and 16H light treatment. A total of 525 metabolites were detected, which were divided into 12 groups, including 124 flavonoids, 89 phenolic acids, 78 lipids, 73 amino acids and their derivatives, 40 nucleotides and their derivatives, 36 organic acids, 16 alkaloids, nine lignans and coumarin, three tannins, one quinone, one terpenoid, and 55 sugars and other metabolites (Table S1). Of these metabolites, seven metabolites existed only in wild type, including pinoresinol, tiliroside, p-coumaroylferuloyltartaric acid, apigenin 8-C-pentoside, psoralenol, maltol and 6-C-Hexosyl luteolin O-pentoside. Five metabolites existed only in *sub-1* mutant, including octadecatetraenoic acid, syringic acid, phthalic acid, hexadecyl ethanolamine and lysoPC (16:1). The principal component analysis (PCA) of differentially accumulated metabolites showed that the value of PC1 was 59.32%, and PC2 was 10.65%. The value of PC1 was much higher than PC2, indicating that genetic background plays an important role in metabolite accumulation, followed by light treatment (Figure 4a). Based on the selection criteria |log2 FC| ≥ 1 and VIP ≥ 1, 221 differentially accumulated metabolites were detected in the OH group, of which 101 down-regulated, 120 up-regulated (Figure 4b, Table S2). Flavonoids accounted for the largest proportion, among which, three main flavones in duckweed decreased significantly including luteolin-6,8-di-C-glucoside (FC: 0.31), apigenin (FC: 0.29), and luteolin (FC: 0.24). Alkaloids (8/8) showed excessive accumulation, including N-Feruloyl tryptamine (FC: 705), N-Feruloyl serotonin (FC: 272), serotonin (FC: 142), tryptamine (FC: 126), N-Trans-feruloyltyramine (FC: 62), N-Cis-feruloyltyramine (FC: 56), Methoxy-N-Caffeoyltyramine (FC: 31) and 6-Deoxyfagomine (FC: 3) (Figure S2, Table S3). In terms of primary metabolites, panose, D-(+)-melezitose, D-glucose and D-(+)-glucose accumulated significantly (Table 4), 42 amino acids (42/44) were significantly increased, including citrulline, proline, arginine, glutamine, lysine, serine, histidine, tyrosine, valine, methionine, threonine, leucine and isoleucine (Figure 4d).

A total of 219 differentially accumulated metabolites were detected in the 16H group, including 97 down-regulated and 122 up-regulated (Figure 4b, Table S2). Flavonoids still accounted for the largest proportion (55/219, 14 up-regulated and 41 down-regulated). Two main flavones were significantly decreased, including apigenin-6,8-di-C-glucoside (FC: 0.50) and luteolin-6,8-di-C-glucoside (FC: 0.31). Alkaloids (7/7) showed excessive accumulation, including N-feruloyl tryptamine (FC: 3847) and N-feruloyl serotonin (FC: 310), tryptamine (FC: 183), serotonin (FC: 149), N-trans-feruloyltyramine (FC: 55), N-cis-feruloyltyramine (FC: 47) and methoxy-N-caffeoyltyramine (FC: 25) (Figure S2, Table S3). In terms of primary metabolites, three carbohydrates (panose, D-(+)-melezitose and D-glucose) and 40 amino acids (40/42) were accumulated significantly, including citrulline, proline, arginine, glutamine, lysine, serine, histidine, tyrosine, valine, methionine and threonine (Figure 4d, Table 4). The Venn diagram suggested that 188 differentially accumulated metabolites existed in both the 0H and 16H groups (Figure 4c). Thirty-three metabolites existed only in the 0H group, including D-(+)-glucose (mws0198), 11-octadecanoic acid (mws2623), 13-HOTrE(r) (pmb2792), and so on. Thirty-one metabolites existed only in the 16H group, including 1-stearoyl-sn-glycero-3-phosphocholine (mws0126), lysoPC (18:0) (pmp001286), and so on. Taken together, the content of sugars, amino acids and alkaloids significantly increased, and flavonoids significantly decreased in *sub-1* mutant.

### 2.7. Transcriptome Analysis

**Figure 5:**
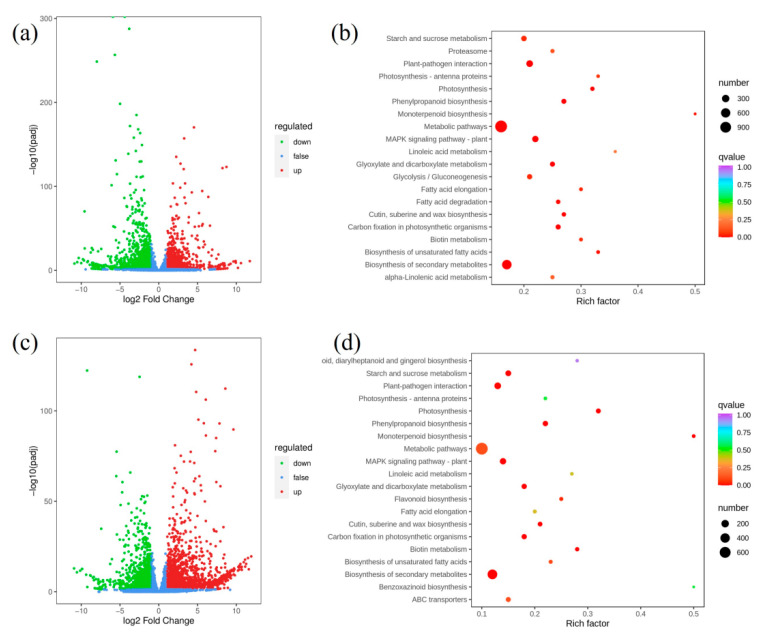
Enrichment analysis of differentially expressed genes. (**a**,**c**) Treatment with 0H light, the volcano map and KEGG enrichment analysis between wild type and *sub-1* mutant. (**b**,**d**) Treatment with 16H light, the volcano map and KEGG enrichment analysis between wild type and the *sub-1* mutant.

In order to investigate the molecular mechanism of starch accumulation in *sub-1* mutant, transcriptome sequencing was performed. The volume of data reached 6.17–10.71 G, Q20 and Q30 of all samples were above 96.74% and 91.55%, respectively, and GC content was between 53.26–55.00% (Table S4). Therefore, the volume and quality of data meet the requirements of bioinformatics analysis. A total of 76,791 unigenes were obtained, among which 43,356 unigenes could be annotated in KEGG, NR, SwissProt, Trembl, KOG, GO and Pfam, accounting for 56.46% of the total unigenes (Table S5). 3297 up-regulated and 3256 down-regulated genes were detected in the 0H group (Figure 5a, Table S6). 31 metabolic pathways were significantly enriched, including starch and sucrose metabolism (109), glycolysis/gluconeogenesis (90), carbon fixation in photosynthetic organisms (81), phenylpropanoid biosynthesis (75) and so on (Figure 5b, Table S7). In the starch metabolism, up-regulated genes include glucose-6-phosphate isomerase (cluster-8142.17850), sucrose synthase (cluster-8142.37261, cluster-8142.42089), glycogen synthase (cluster-8142.7837) and isoamylase (cluster-8142.28410), and so on. In the 16H group, a total of 2181 up-regulated and 2009 down-regulated genes were detected (Figure 5c, Table S6). 24 metabolic pathways were significantly enriched (*p* < 0.05), including photosynthesis (42), phenylpropanoid biosynthesis (61), carbon fixation in photosynthetic organisms (56), starch and sucrose metabolism (83) and so on (Figure 5d, Table S7). In the starch and sucrose metabolism, up-regulated genes include granule-bound starch synthase (cluster-8142.30997, cluster-8142.31500), starch synthase (cluster-8142.35463), phosphoglucomutase (cluster-8142.29861) and hexokinase (cluster-8142.15573, cluster-8142.18958), and so on. 

Transcription factors (TFs) play an important role in gene expression regulation. It reported that WRKY, AP2, NAC and bZIP were involved in starch metabolism [27,28]. In this project, a total of 215 up-regulated and 183 down-regulated TFs were detected in the 0H group, including 40 AP2/ERF-ERF, 29 WRKY, 26 bHLH, 21 bZIP, 18 NAC, 16 MYB, and so on (Table S8). In the 16H group, 147 up-regulated and 109 down-regulated TFs were detected, including 33 AP2/ERF-ERF, 21 WRKY, 16 NAC, 13 MYB, 11 bZIP, 10 bHLH, and so on (Table S8). These differentially expressed transcription factors provide valuable genetic resources for the transcriptional regulation of the efficient accumulation of starch in duckweed.

### 2.8. Enzyme Genes in Starch and Sucrose Metabolic Pathways Verified by Q-PCR 

**Figure 6:**
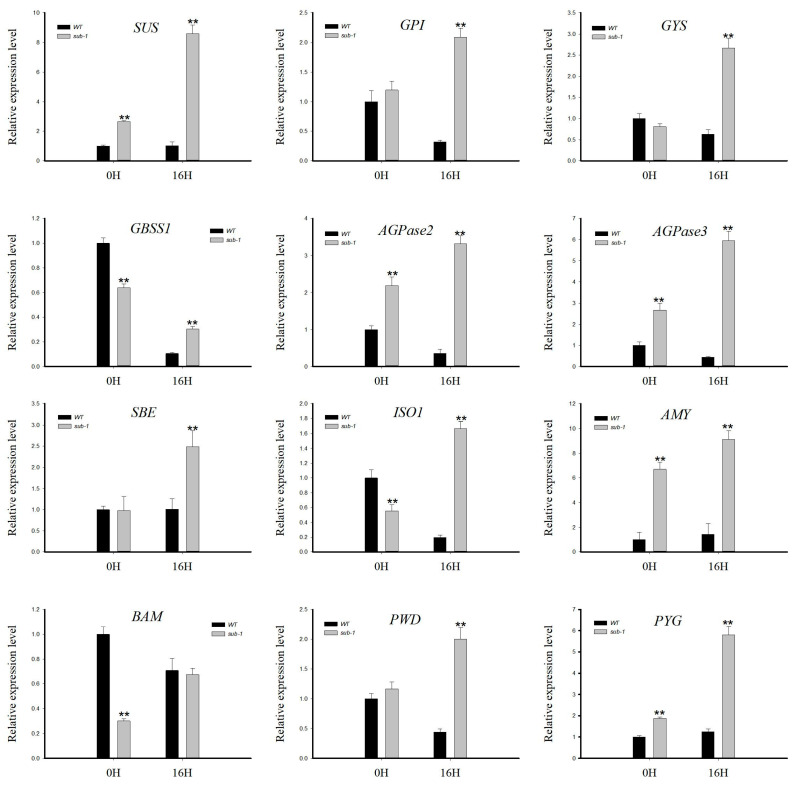
Transcriptional expression analysis of key enzyme genes in starch and sucrose metabolic pathways. *SUS*: sucrose synthase, *GPI*: glucose-6-phosphate isomerase, *GYS*: glycogen synthase, *GBSS*: granule-bound starch synthase, *ADPase2*: ADP-glucose pyrophosphorylase 2, *ADPase3*: ADP-glucose pyrophosphorylase 3, *SBE*: starch branching enzyme, *ISA1*: isoamylase 1, *AMY*: α-amylase, *BAM*: β-amylase, *PWD*: phosphoglucan-water dikinase, *PYG*: glycogen phosphorylase. Bars represent standard deviations (SD) from three biological replicates. Asterisks indicate the significant difference between wild type and *sub-1* mutant (** *p* < 0.01) using Duncan’s test.

**Figure 7:**
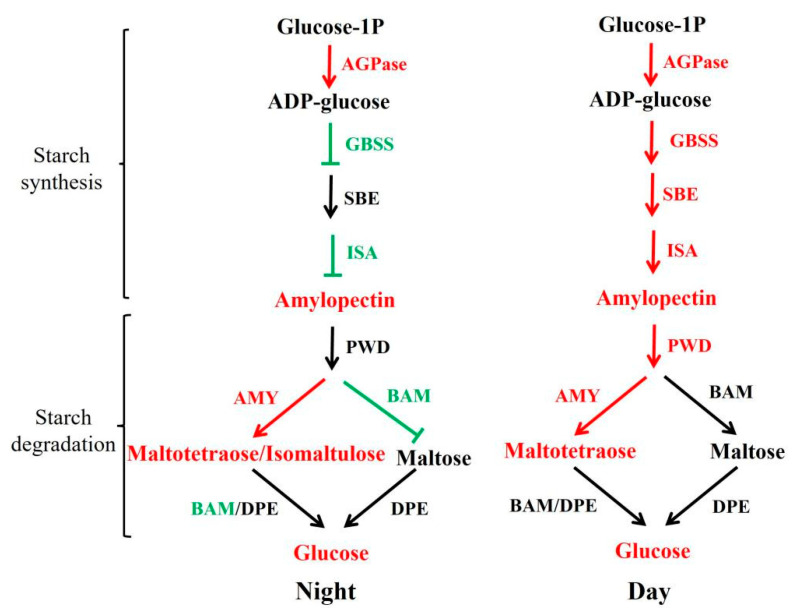
Reconstruction of the starch biosynthetic pathway with differentially expressed genes and metabolites in *sub-1* mutant. green represent down-regulated metabolites and genes, red represent up-regulated metabolites and genes. In addition, the red arrow represents boost, the black arrow represents no difference, and T represents inhibition.

The mRNA of wild type and *sub-1* mutant were extracted and then reverse transcribed into cDNA. The transcriptional levels of genes were analyzed using 18S RNA as an internal reference gene. Those detected genes contain sucrose synthase (*SUS*), glucose-6-phosphate isomerase (*GPI*), glycogen synthase (*GYS*), granule-bound starch synthase 1 (*GBSS1*), ADP-glucose pyrophosphorylase 2 (*AGPase2*), ADP-glucose pyrophosphorylase 3 (*AGPase3*), starch branching enzyme (*SBE*), isoamylase1 (*ISA1*), α-amylase (*AMY*), β-amylase (*BAM*), phosphoglucan-water dikinase (*PWD*) and glycogen phosphorylase (*PYG*). In the 0H group, *SUS*, *AGPase2*, *AGPase3*, *AMY* and *PYG* genes were up-regulated, while *GBSS1*, *ISO1* and *BAM* genes were down-regulated (Figure 6 and Figure 7). In the 16H group, *SUS*, *GPI*, *GYS*, *GBSS1*, *AGPase2*, *AGPase3*, *SBE*, *ISO1*, *AMY*, *PWD* and *PYG* genes were up-regulated, and the *BAM* gene showed no difference in *sub-1* mutant (Figure 6 and Figure 7).

## 3. Discussion

At present, wild-type duckweed with high starch is relatively few, and it is particularly important to create high-starch germplasm by radiation mutagenesis or genetic engineering. In the previous study, we screened a new high-starch variety *sub-1*, whose starch content was more than twice that of wild type. In this study, we found that the proportion of amylopectin in *sub-1* mutant was as high as 84.04%, and degree of branching was significantly increased, while the crystallinity was significantly decreased. In the metabolome, a total of 525 metabolites were detected, among which contents of glucose, amino acids and alkaloids were significantly increased, and flavonoids were significantly decreased. The key enzyme genes (*SUS*, *GPI*, *GYS*, *GBSS1*, *AGPase2*, *AGPase3*, *SBE*, *ISA1*, *AMY*, *BAM*, *PWD*, and *PYG*) in the sucrose and starch metabolism were differentially expressed in *sub-1* mutant. This study provided a new idea for the creation of crop germplasm with high-amylopectin. 

The fronds of duckweed (*L. aequinoctialis* 6002) used in this study are inverted oval (Figure 1a). However, the fronds of *sub-1* mutant were square and thicker, especially with an extra layer of aerenchyma (Figure 1b). These changes in the structure of fronds may have a great impact on photosynthesis, which in turn affects carbon fixation and starch accumulation [11]. I_2_/KI and PAS staining showed that large amounts of sugars and starch were accumulated in the fronds of *sub-1* mutant, but starch granules were not found in the upper epidermal cells (Figure 1). These results indicated that upper epidermal cells of *sub-1* mutant play an important role in the sucrose metabolism, but do not participate in starch accumulation. It is well known that amylose turns blue when exposed to iodine and amylopectin turns reddish-brown when exposed to iodine. There may be two reasons for both the reddish-brown of the wild type and *sub-1* mutant starch when exposed to iodine in our research. First, there is less sample on the paraffin section, which leads to low starch granules. Second, duckweed has a higher proportion of amylopectin. Starch granules of duckweed were extracted to detect the amylopectin content. The content of amylopectin in the wild type was 75.66%, and that of the *sub-1* mutant was 84.04% (Table 1). This result was consistent with our previous report [12]. In natural starch, the amylopectin content was generally 72–85% in starch granules, which varied depending on species, growth environment and developmental stage [2,3]. Previous report of our laboratory showed that the amylopectin content of two varieties starch were 72.23% (*Lemna minor*) and 79.15% (*Spirodela oligorrhiza*), respectively [10]. Therefore, the *sub-1* mutant is a new germplasm resource with high amylopectin. 

In general, amylose has a certain number of long-chain branches with a low branching degree, while amylopectin has a large number of short-chain branches with a high branching degree. Compared with wild type, the branching degree of *sub-1* mutant starch was significantly increased by 19.6%, which further proved that the *sub-1* mutant had a higher amylopectin content (Table 3). The shape of the chain length distribution curves of amylopectin were highly matched between the two varieties (Figure 2), showing two valleys at positions DP 6 and DP 36, and two peaks at DP 13 and DP 46, which were consistent with the chain length distribution of amylopectin from other species [10]. The average chain length of wild-type and *sub-1* mutant was DP 23.2 and DP 22.7 (Table 2), respectively, which was higher than the chain length of potato (DP 20.5), cassava (DP 19.1–19.5) and maize (DP 21.3) [29,30,31], but shorter than the average chain length of *S. oligorrhiza* (DP 28.5) and *L. minor* (DP 28.2) [10]. The proportion of amylopectin affects the physicochemical properties of starch granules, such as swelling power and water solubility [32]. Studies have shown that swelling power was negatively correlated with the amylose content [25]. Swelling power of maize starch was negatively correlated with the propotion of long chain of amylopection [33]. In this study, swelling power of *sub-1* mutant starch was significantly increased by 97.07%, and water solubility was decreased by 29.42% (Table 3). Overall, these results indicated that swelling power was positively correlated with amylopectin content, especially the medium and long chain of amylopectin (DP 13–24), and negatively correlated with short chain (DP 6–12) and long chain (DP > 36) amylopection in *sub-1* mutant (Table 2).

Previous studies have shown that the amylose of potato, banana, maize and cassava were semi-crystalline V-type [34]. Wild-type starch used in this study also have typical V-type structural characteristics, which means that the wild-type starch forms complexes with fatty acids, alcohols, and other components. However, the starch granules of *sub-1* mutant showed B-type structural characteristics. It has been reported that the crystal region of starch was composed of double helices wrapped by short chains amylopectin and small crystals packed into them [35]. The crystallinity of *sub-1* mutant starch was only 8.94%, which was much lower than that of the wild type (22.3%) (Figure 3), probably due to the reduced porportion of short-chain amylopectin (DP 6–12). 

Light plays an important role in starch synthesis and degradation in plants. It is unknown whether wild type and *sub-1* mutant differ in their response to circadian rhythm. Treatment with 0H and 16H light exposure, the samples were collected for transcriptome and metabolome analysis. Principal component analysis (PCA) showed that the value of PC1 (59.32%) was much higher than that of PC2 (10.65%) (Figure 4a). In addition, 188 differentially accumulated metabolites (DAMs) were existed in both groups, 33 DAMs were only existed in the 0H group, and 31 DAMs were only existed in the 16H group (Figure 4c). Based on the above results, the difference in the genetic background between wild type and *sub-1* mutant is the main factor responsible for the difference in metabolites, and circadian rhythm is a secondary factor. During the process of starch degradation, α-amylase hydrolyzes starch to malto-oligo saccharides, while β-amylase hydrolyzes starch to maltose to produce glucose [36,37]. In the 0H group, maltotetraose and isomaltulose were up-regulated by 1.30-fold and 1.24-fold respectively. In the 16H group, maltotetraose was up-regulated 1.61-fold. However, there was no change in maltose accumulation in this study. The results of qRT-PCR showed that the expression of the *AMY* gene was significantly up-regulated by more than six-fold in the 0H and 16H treatment groups (Figure 6). While the *BAM* expression was down-regulated by about 70% in the 0H group, and there was no significant change in the 16H group (Figure 6). The expression of *AMY* and *BAM* in transcriptome was consistent with the results of qRT-PCR. Thus, degration of starch into glucose is mainly dependent on *AMY* rather than *BAM* in *sub-1* mutant (Figure 7). Under physiological conditions, AtBAM3 is a core enzyme and is involved in the degradation of transient starch at night [38]. Under osmotic stress, AtBAM1 becomes the main starch degrading enzyme and participates in diurnal/transitory starch degradation in guard cells and epidermal cells [38,39]. In our previous study, the starch turnover was decreased in the *sub-1* mutant [10]. Consequently, these results suggested that down-regulated expression of *BAM* gene suppresses the degradation of transitory starch, which leads to the starch accumulation in the *sub-1* mutant (Figure 7).

In the 0H and 16H light groups, differentially expressed genes (DEGs) of starch and sucrose metabolic pathways were significantly enriched (Figure 5). Therefore, qRT-PCR was used to analyze the transcription level of DEGs. In the *sub-1* mutant, *SUS*, *PYG*, *GPI*, *GYS* and *PWD* were up-regulated, and these genes enhance glycogen synthesis and accumulation (Figure 6). At the same time, panose, melezitose and glucose were accumulated in the *sub-1* mutant, which was an important reason for the deep purple-red of PAS staining (Figure 1). ADP-glucose pyrophosphorylase (ADPase) is the first rate-limiting enzyme in the starch synthesis pathway, and the up-regulated expression of *AGPase2* and *AGPase3* in *sub-1* mutant provides sufficient ADP-glucose for starch synthesis (Figure 7). Granule bound starch synthase (GBSS) transfers the glucose group of ADP-glucose to the non-reduced end of the (1, 4) -glucose chain, forming an α-(1, 4) glycosidic bond. In the 0H treatment group, *GBSS1* gene was significantly down-regulated and further decreased after 16 h of light treatment, indicating that the synthesis of amylose was inhibited in the *sub-1* mutant (Figure 6 and Figure 7). Starch branching enzyme (SBE) catalyzes the cleavage of 1, 4-glucoside bond to form 1, 6-glucoside bond and produce the branched chain of starch. Under light conditions, up-regulated of *SBE* gene was closely related to the increase of amylopectin content in the *sub-1* mutant (Figure 6 and Figure 7). 

## 4. Materials and Methods

### 4.1. Materials and Culture Environment

The wild-type duckweed (*L. aequinoctialis* 6002) used in this study was collected from Weishan Lake, Shandong, China (latitude and longitude: 116.98, 34.94). A new duckweed variety *sub-1* was obtained by differentiation and screening of callus (*L. aequinoctialis* 6002) induced by heavy ion radiation [11]. Both wild type and *sub-1* plants were cultured in liquid SH medium with 10 g/L sucrose. The parameters of culture room were set as follows: 25 ± 1 °C temperature, 16 h/8 h (day/night), 80 μmol m^−2^s^−1^ light intensity, 50–60% relative humidity.

### 4.2. I_2_/KI Staining

Duckweed was placed in 4% paraformaldehyde solution, then dehydrated, impregnated and fixed with a series of concentrations of ethanol. The sample was impregnated with different proportions of xylene and absolute ethanol for 45 min at each concentration. Impregnated with xylene: Paraffin (1:3, 1:1, and 0:1) for 12 h. Paraffin and sample were added to the embedding molds and removed after cooling on ice. The thickness of slice was set to 8 um, and slices were cut and placed in water at 42 °C. Slices were fully unfolded and placed on the slide. The dried slides were placed in a dye tank filled with 100% xylene for 30 min, and deparaffinized with serial concentrations of alcohol. The plant materials were stained with 1% I_2_/KI staining solution for 30 min and then washed with purified water. Slices were observed and photographed using a microscope (Axio Scope A1, ZEISS, Suzhou, China).

### 4.3. Periodic Acid-Schiff (PAS) Staining 

Slices were deparaffinized as described in the I_2_/KI staining. Then the slices were stained in solution B (0.5% periodate acid) for 15 min, and washed by purified water for 10 s. Then slices were stained with solution A (Schiff reagent) in the dark for 30 min and washed with purified water for 5 min. Finally, slices were stained with solution C (Hematosylin) for 30 s and washed with water. Microscope (Axio Scope A1, ZEISS, Suzhou, China) observed the slices and photographed.

### 4.4. Extraction of Starch Granules

Duckweed was inoculated into 40 cm × 60 cm iron plates and cultured for 2 weeks. The plant materials were broken using blender, and the residue was filtered through filter cloth. The mixture of water and starch granules were centrifuged at 5000× *g* for 5 min and discarded the supernatant. The starch granules were dried at 37 °C. It can be used for the determination of amylose content, branching degree, chain length distribution, swelling power, X-ray differaction (XRD) and fourier transformed infrared spectrometry (FTIR) analysis.

### 4.5. Determination of Amylose Content

About 10 mg starch granules were taken and added 100 μL 95% alcohol and 900 μL NaOH solution. The mixture was boiled for 13 min, then the volume was fixed to 10 mL. Mix 0.5 mL supernatant, 0.1 mL acetic acid and 0.2 mL potassium iodide solution, and the volume was fixed to 10 mL and placed in the dark for 10 min. The absorption value was measured at 620 nm [40]. According to the standard curve Y = 215.71X − 15.108, the content of amylose was calculated.

### 4.6. Degree of Branching 

About 5 mg starch granules were taken, and added 1 mL DMSO, heated at 80 °C overnight. After centrifugation at 12,000 rpm for 10 min, the supernatant was added to the tube for nuclear magnetic analysis. The instrument used in this experiment was Bruker BioSpin GmbH. The scanning times were 32, resonant RF was 500.23 MHz, and NMR spectrum was 1 H [41].

### 4.7. Chain Length Distribution of Amylopectin

The starch granules were digested with isoamylase (Sigma-Aldrich, Shanghai, China), and the supernatant of the sample was taken and used for the analysis of chain length distribution of amylopectin [42]. Thermo ICS5000 ion chromatography system (ICS500+, Thermo Fisher Scientific, Waltham, MA, USA) and Dionex^TM^ CarboPac^TM^ PA200 (250 × 4.0 mm, 10 μM) chromatographic column were used. Mobile phase A: 0.2 M NaOH, mobile phase B: 0.2 M NaOH/0.2 M NaAC, the flow rate was 0.4 mL/min, column temperature was 30 °C. The composition of enzymatic hydrolysate was analyzed by electrochemical detector.

### 4.8. X-ray Differaction (XRD) Analysis

After water balance, starch granules were screened with 100 mesh, placed on the loading platform, spread evenly, pressed tightly, and tested on the machine. X’Pert Pro X-ray diffractometer (X’pert PRO, PANalytical, Alemlo, The Netherlands) was used in this experiment. The parameters: copper target Cu Kα (λ = 0.15406 nm), power 1600 W (40 kV × 40 mA), NaI scintillation counter. The diffraction angle (2θ) was in the range of 4°–60° with a scanning rate of 4°/min and step size 0.02° [43]. DS-SS-RS settings were 0.5 mm–0.25 mm–0.1 mm. The data were calculated using MDI Jade 5.0 software (Materials Data Inc., Livermore, CA, USA). The crystallinity, crystal morphology and diffraction peaks at 2θ value (angle) were calculated in the starch sample, respectively.

### 4.9. Fourier Transformed Infrared Spectrometry (FTIR) Analysis

First, starch granules were screened with 100 mesh. A small amount of sample was mixed with potassium bromide and pressed into tablets. Fourier transformed infrared spectrometry Nicolet iZ-10 was used (Thermo Fisher Scientific, Inc., Waltham, MA, USA). The spectra were set from 400 cm^−1^ to 4000 cm^−1^, the number of scans was 32, and the resolution was 4.00 cm^−1^ [43].

### 4.10. Water Solubility (WS) and Swelling Power (SP)

Mix 0.1 g starch and 10 mL deionized water in the tube (M1), water bath at 95 °C for 60 min. Cool the tube to room temperature and centrifuge at 8000× *g* for 20 min. The tube with precipitation was dried at 80 °C for 20 min, and put into a incubator for 20 min, marked as M2. The weight of aluminum drum was marked M3. The supernatant in the aluminum drum was placed in the oven at 80 °C to a constant weight, marked as M4 [44]. The calculation formula of water solubility and swelling power were as follows:

WS (%) = [(M4 − M3)/M] × 100

SP (g/g) = (M2 − M1)/[M × (1 − WS)]

### 4.11. Metabolome and Transcriptome Analysis

Wild type and *sub-1* mutant were exposed to light for 0H and 16H, respectively, and total RNA was extracted from 12 samples. The cDNA library was constructed with high quality RNA and quantified by Qubit 2.0. The insert size of the cDNA library was detected by Agilent 2100. The effective concentration of the library was quantitatively detected by Q-PCR. After being qualified, sequencing was performed on the illumina HisSeq platform. Comparison of clean data with rice genome, the mapped data was obtained. Based on the number of reads assembled genome, the FPKM value and differential expression of each gene were calculated. The filter condition of differentially expressed genes (DEGs) were as follows: |log2Fold Change| ≥ 1 and FDR < 0.05. Finally, the enrichment analysis of DEGs were carried out, such as Kyoto Encyclopedia of Genes and Genomes (KEGG), Gene Ontology (GO) and Clusters of Orthologous Groups (KOG).

Similar to the transcriptome sampling, a total of 12 samples were used for metabolome and divided into 4 groups with 3 biological replicates. Metabolite detection was based on UPLC-MS/MS (UPLC: Shim-pack UFLC SHIMADZU CBM30A, MS/MS: Applied Biosystems 6500 QTRAP) data collection system. The original data was compressed into multiple principal components to describe the features of original data. PC1 represents the most obvious features, PC2 represents the most significant features except for PC1. The heatmap was drawn by the pheatmap package of the R software, which exhibits the accumulation pattern of metabolites among 12 samples. The screening conditions of differentially accumulated metabolites (DAMs) were as follows: Fold change ≥ 2 or fold change ≤ 0.5, meanwhile, VIP ≥ 1.

### 4.12. Differentially Expressed Genes Verified by Quantitative Real-Time PCR (Q-PCR)

Total RNA was extracted after light treatment for 0H and 16H, respectively. The mRNA was reverse transcribed into cDNA by First-Strand cDNA Synthesis SuperMix (TransGen, Beijing, China), and the product could be used as the template of Q-PCR. The reaction system was as follows: 1 μL template, 0.5 μL forward primer, 0.5 μL reverse primer, 10 μL SYBR Green, 8 μL ddH_2_O. The procedure of the Q-PCR detection system (QuantStudio 1, ABI, Waltham, MA, USA) was as follows: 98 °C predenature, 94 °C denature, 74 °C anneal and extend. 18S rRNA was used as the housekeeping gene. The expression of candidate genes was obtained by calculating the 2^−ΔΔCT^ value.

### 4.13. Statistical Analysis

At least 3 biological replicates were used for statistical analysis, and the results were expressed as mean ± standard deviation. Duncan’s multiple test of SPSS 17.0 (SPSS Inc., Chicago, IL, USA) (*p* < 0.05) and data processing system (DPS, Zhejiang University, Zhejiang, China) were used. The asterisk means that there is a significant difference between wild type and *sub-1* mutant (* *p* < 0.05, ** *p* < 0.01). The letter in Table 1, Table 2 and Table 3 represent significant differences between the wild type and *sub-1* mutant. 

## 5. Conclusions

The *sub-1* mutant is a new duckweed variety with high amylopectin, and its amylopectin content is up to 84.04%. At the same time, the degree of starch branching increased by 19.6% and the number of branched chain increased. These traits are benefit for the enzymatic hydrolysis of starch and bioethanol conversion. Up-regulated expression of *AGPase2*, *AGPase3* and *SBE* and down-regulated *GBSS1* are the important reasons for the decreased ratio of amylose/amylopectin (Figure 7). In addition, the accumulation of malto-oligo saccharides and glucose in the *sub-1* mutant implies that the up-regulated *AMY* gene plays a major role in the starch degradation (Figure 7). While the down-regulated expression of *BAM* gene at night leads to a decrease in starch degradation and the underlying molecular mechanism needs further investigation. This study laid a foundation for the development and utilization of the *sub-1* mutant starch and provided theoretical guidance for the germplasm creation of high amylopectin crops.

## Figures and Tables

**Table 1:** The proportion of amylopectin in the starch of wild type and *sub-1* mutant.

Starch Charecteristics	Species	
	Wild Type	*sub-1*
Amylose (%)	24.34 ± 0.26 a	15.96 ± 0.15 b
Amylopectin (%)	75.66 ± 0.26 b	84.04 ± 0.15 a
Amylose/Amylopectin	0.32 ± 0.01 a	0.19 ± 0.01 b

Note: Different letters indicate the significant difference between wild type and *sub-1* mutant (*p* < 0.05) using Duncan’s test.

**Table 2:** Chain length distributions of the amylopectin obtained from HPAEC-PAD.

Sample	DP 6–12 (%)	DP 13–24(%)	DP 25–36(%)	DP > 36 (%)	DP
WT	21.26 ± 0.06 a	47.30 ± 0.06 b	14.02 ± 0.03 a	17.42 ± 0.08 a	23.16 ± 0.04 a
*sub-1*	20.78 ± 0.06 b	49.50 ± 0.16 a	13.02 ± 0.02 b	16.70 ± 0.19 b	22.72 ± 0.08 b

Note: Means with different letters differ significantly between wild type and *sub-1* mutant (Duncan’s test, *p <* 0.05).

**Table 3:** Water solubility, swelling power and branching degree analysis.

Sample	WS	SP	DB
WT	33.96 ± 1.07 a	10.24 ± 0.47 b	6.67 ± 0.02 b
*sub-1*	23.97 ± 1.48 b	20.18 ± 0.33 a	7.98 ± 0.06 a

Note: Means with different letters differ significantly between wild type and *sub-1* mutant (Duncan’s test, *p* < 0.05). WS: Water solubility, SP: Swelling power, DB: Degree of branching.

**Table 4:** Differentially accumulated carbohydrate compounds.

	Compound	VIP	Fold Change	Type
CK vs. *sub-1* (0H)	Panose	1.14	5.48	Up-regulated
	D-(+)-Melezitose	1.14	3.38	Up-regulated
	D-Glucose	1.15	2.61	Up-regulated
	D-(+)-Glucose	1.15	2.32	Up-regulated
CK vs. *sub-1* (16H)	Panose	1.16	2.65	Up-regulated
	D-(+)-Melezitose	1.13	2.29	Up-regulated
	D-Glucose	1.13	2.23	Up-regulated

## Data Availability

The raw data of transcriptome have been deposited in the Sequence Read Archive (SRA) of National Center for Biotechnology Information (NCBI, https://www.ncbi.nlm.nih.gov/) under project accession number PRJNA906249.

## Supplementary Materials

The following supporting information can be downloaded at: https://www.mdpi.com/article/10.3390/ijms24032934/s1.
