# Supplementary material for: Efficient Accumulation of Amylopectin and Its Molecular Mechanism in the Submerged Duckweed Mutant
Source: Int J Mol Sci. 2023 Feb 2;24(3):2934. doi: 10.3390/ijms24032934 (PMC9917893; doi:10.3390/ijms24032934)
Supplement: Supplementary file 1 [file ijms-24-02934-s001.zip › Figures S1 and S2.pdf]

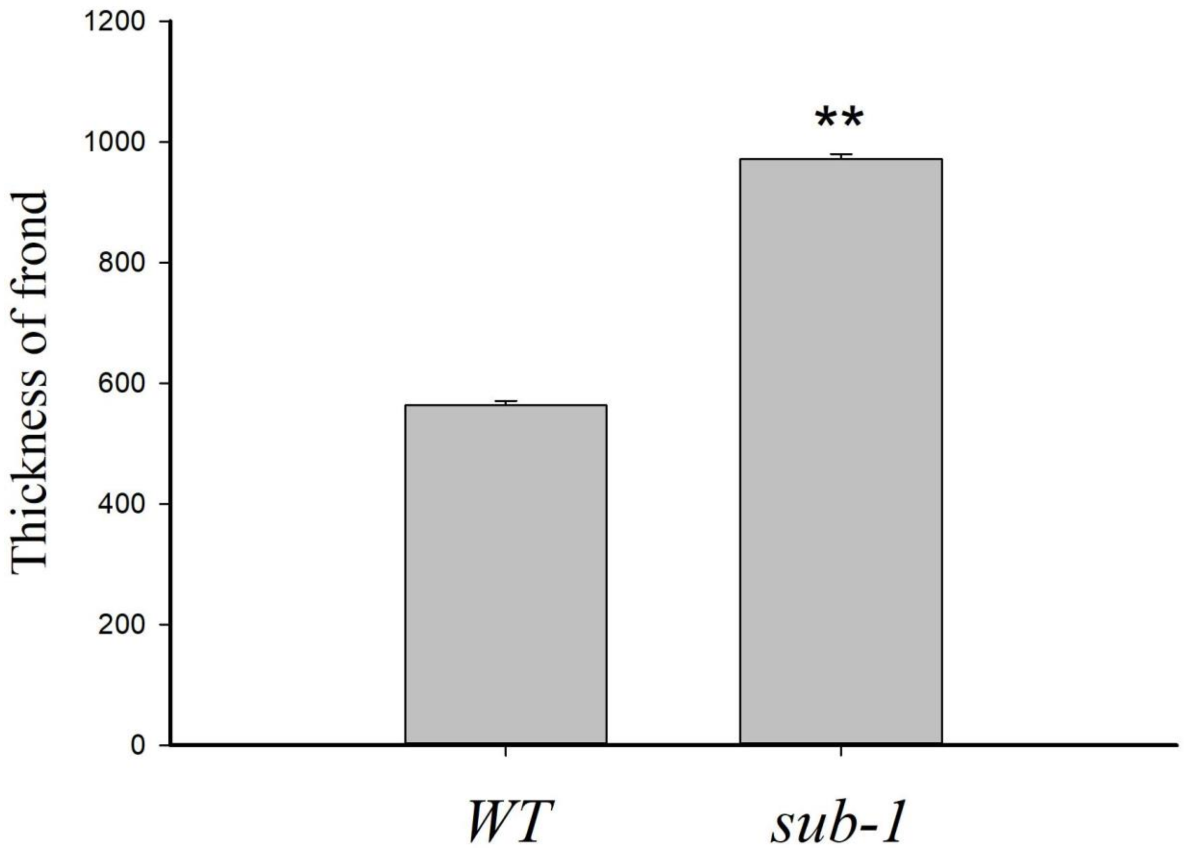

Figure S1. Statistical analysis of fronds thickness.

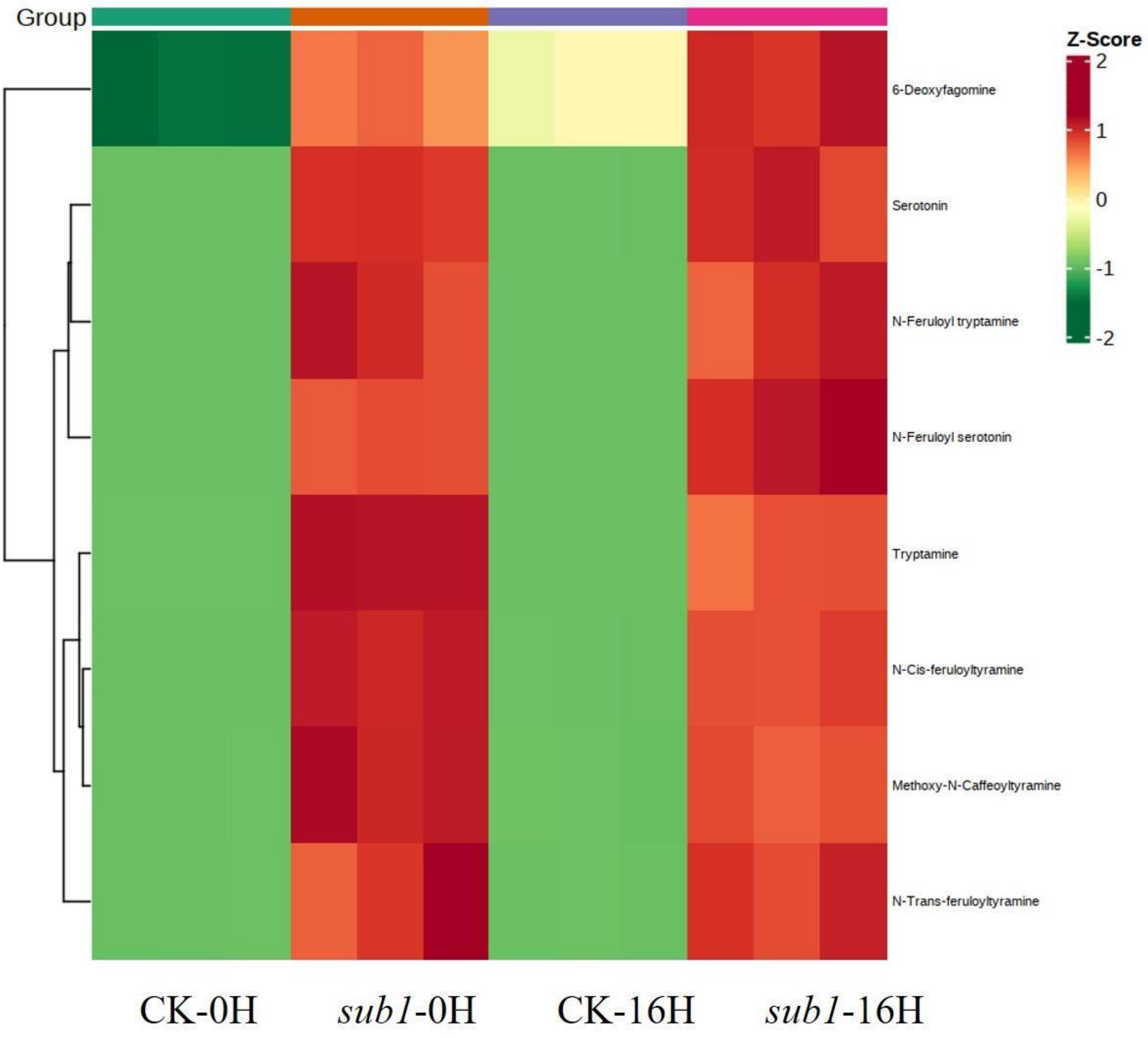

Figure S2. Heatmap analysis of differentially accumulated alkaloids.
