# Supplementary material for: Efficient Accumulation of Amylopectin and Its Molecular Mechanism in the Submerged Duckweed Mutant
Source: Int J Mol Sci. 2023 Feb 2;24(3):2934. doi: 10.3390/ijms24032934 (PMC9917893; doi:10.3390/ijms24032934)
Supplement: Supplementary file 1 [file ijms-24-02934-s001.zip › Table S4 The volume and quality of transcriptome data.pdf]

Table S4 The volume and quality of transcriptome data

Sample	Raw Reads	Clean Reads	Clean Base(G)	Q20(%)	Q30(%)	GC Content(%)
CK0H-1	64220158	62220800	9.33	97.33	92.78	54.5
CK0H-2	62516028	60069648	9.01	97.44	92.96	54.56
CK0H-3	59999518	57498620	8.62	97.3	92.69	54.47
CK16H-1	49364072	46936874	7.04	97.54	93.15	53.95
CK16H-2	46918504	44699878	6.7	97.3	92.74	55
CK16H-3	61602486	58721656	8.81	97.3	92.66	53.95
FS75-0H-1	57955930	55653916	8.35	97.21	92.48	53.65
FS75-0H-2	48313500	46606576	6.99	97.33	92.76	53.93
FS75-0H-3	54365540	52324436	7.85	96.99	92.01	53.85
FS75-16H-1	43334904	41115290	6.17	96.74	91.55	53.26
FS75-16H-2	74823186	71417328	10.71	97.37	92.78	53.39
