# Supplementary material for: Efficient Accumulation of Amylopectin and Its Molecular Mechanism in the Submerged Duckweed Mutant
Source: Int J Mol Sci. 2023 Feb 2;24(3):2934. doi: 10.3390/ijms24032934 (PMC9917893; doi:10.3390/ijms24032934)
Supplement: Supplementary file 1 [file ijms-24-02934-s001.zip › Table S5 All genes annotated to the public database.pdf]

Table S5 All genes annotated to the public database

Database	Number of Genes	Percentage (%)
KEGG	31006	40.38
NR	41223	53.68
SwissProt	29262	38.11
Trembl	41313	53.80
KOG	25753	33.54
GO	35297	45.97
Pfam	32362	42.14
Annotated in at least one Database	43356	56.46
Total Unigenes	76791	100.00
